# Updates, Controversies, and Emerging Approaches in Colorectal Screening

**DOI:** 10.7759/cureus.17844

**Published:** 2021-09-09

**Authors:** Tiffany Wang, Tyler Tsang, Alla Turshudzhyan, Heather Dacus, Micheal Tadros

**Affiliations:** 1 Internal Medicine, Albany Medical College, Albany, USA; 2 Internal Medicine, University of Connecticut, Farmington, USA; 3 Internal Medicine, New York State Department of Health, New York, USA; 4 Gastroenterology and Hepatology, Albany Medical Center, Albany, USA

**Keywords:** colorectal cancer, cancer screening, mass screening, surveillance, colonoscopy, quality indicators

## Abstract

Colorectal cancer (CRC) is the third most common cancer worldwide and the second leading cause of cancer-related deaths. Despite the threatening statistics, the US burden for CRC has been decreasing, which is likely multifactorial and has partial contribution from widespread timely screening, more advanced CRC treatment, and daily aspirin use in some patients. While overall death rate from CRC decreased by approximately a half between 1975 and 2012, epidemiologic studies demonstrate that CRC incidence is increasing in the younger population. This pattern has prompted the American Cancer Society (ACS) to revise their guidelines. In this review, we plan to discuss the most recent changes in guidelines, data to support them, controversies concerning CRC screening methods, age to start and to stop screening, and post-colonoscopy/polypectomy surveillance guidelines.

## Introduction and background

Colorectal cancer (CRC) ranks third in incidence and second in mortality for cancers worldwide [1]. The most recent data from the US Surveillance, Epidemiology, and End Results (SEER) program projects there to be approximately 148,000 persons to be newly diagnosed with CRC in 2020 in the US with an estimated 53,200 CRC deaths in 2020. In fact, 12% of these will be diagnosed in individuals younger than age 50. Currently the annual CRC incidence rate is 38.7 per 100,000 persons and the mortality rate is 13.9 per 100,000 persons [2].

Despite these threatening statistics, the national burden for CRC has been decreasing. The death rate has decreased by approximately half between 1975 and 2012 [3]. While many attributed this decline to the rapid expansion in screening colonoscopies, some argued that the decline in CRC incidence cannot be fully explained by increases in screening alone. As a result, recent studies have shown a partial contribution from more advanced CRC treatments, reduction in smoked meat consumption, and the use of daily low-dose aspirin [2,4]. Despite the decline in CRC incidence, epidemiologic studies demonstrate that CRC incidence is increasing in the younger population, which has prompted the American Cancer Society (ACS) to revise their guidelines [5].

## Review

CRC screening methods and effectiveness

Stool-Based CRC Screening Tests

Stool-based methods for CRC screening include the guaiac-based fecal occult blood test (gFOBT), fecal immunochemical test (FIT), and multitarget stool DNA test (mt-sDNA, also known as FIT-DNA or Cologuard). Stool-based screening techniques are fast and non-invasive [5]. While stool-based screening is convenient and helps limit the number of patients who need a screening colonoscopy, a positive result requires a timely colonoscopy and can result in loss of patient to follow up [5,6].

gFOBT works by detecting peroxidase activity in the heme portion of hemoglobin [5]. In 2016, a systematic review conducted for the US Preventive Services Task Force (USPSTF) evaluated 419,966 individuals and found a reduction in CRC-specific mortality after two to nine rounds of biennial gFOBT screening when compared to no screening (relative risk=0.78; 95% confidence interval (CI): 0.63 to 0.93 at 30 years) [7]. Additionally, gFOBT demonstrates an adequate sensitivity (62% to 79%) and good specificity (87% to 96%) for detecting CRC [8]. One noteworthy limitation of gFOBT is its ability to generate false positive results with certain foods, vitamins, and medications. gFOBT also requires three consecutive stool samples compared to one sample needed for FIT [9].

FIT is an immunoassay that selectively detects the globin portion of human hemoglobin, a feature that eliminates the need for patients to follow dietary restrictions prior to testing [9]. Since globin is readily degraded by digestive enzymes in the upper GI tract, positive FIT results are more specific for colorectal bleeding than gFOBT [9]. A 2014 meta-analysis determined the pooled sensitivity and specificity of FIT for CRC to be approximately 0.79 (95% CI: 0.69 to 0.86) and 0.94 (95% CI: 0.92 to 0.95), respectively [10]. Although FIT shows improved sensitivity and maintains comparable specificity for CRC as compared to gFOBT, it is limited in detecting advanced adenomas and serrated polyps [11]. Another meta-analysis revealed that FIT had lower false positive and false negative rates when compared to gFOBT (RR -4.06; CI of 95%) [12].

FIT-DNA combines the FIT immunoassay with a molecular assay detecting altered genetic biomarkers. A study conducted with 9989 average-risk participants found an improved sensitivity in the detection of CRC (92% vs. 74% with FIT), advanced precancerous lesions (42% vs. 24% with FIT) and sessile serrated lesions 1cm or larger (42% vs. 5% with FIT). However, the study also found that FIT-DNA had a lower specificity when compared with FIT (86% vs. 95%) [13]. Additionally, the false positive rate of FIT-DNA is 13%, which ultimately results in unnecessary colonoscopies [14].

Direct Visualization CRC Screening Tests

Direct visualization screening methods include colonoscopy, computed tomography colonography (CT colonography), and flexible sigmoidoscopy (FS). Positive results from CT colonography or FS require a follow-up colonoscopy [5].

Colonoscopy is an endoscopic procedure that allows for visualization of the entire colon as well as biopsy and polypectomy if needed [5]. In 2013, a prospective cohort trial involving 88,902 participants found an association between colonoscopy and lower CRC mortality rate when compared with no colonoscopy (hazard ratio (HR)=0.32; 95% CI 0.24-0.45) [15]. The observed reduction in CRC incidence and mortality following colonoscopy was stronger for distal than for proximal cancers, which is likely due to variability in detection of proximal serrated polyps, higher prevalence of flat lesions in proximal colon, poor bowel preparation of proximal colon, and incomplete colonoscopies [16,17].

FS visualizes the distal half of the colon, and it can be performed without sedation [5]. A meta-analysis of FS found that FS was associated with a reduction in CRC-specific mortality at 11 to 12 years of follow-up (incidence rate ratio=0.73; 95% CI 0.66 to 0.82). The effect on mortality was limited to distal cancers (incidence rate ratio=0.63; 95% CI 0.49 to 0.84) [10]. Interestingly, a single FS screening reduces incidence rate of CRC by 34% in men, but not in women [17].

CT colonography is a direct visualization test that is non-invasive and can visualize the entire colon [5]. Lin et al. concluded that CT colonography was comparable to colonoscopy in detecting adenomas 6mm or larger but had a wider degree of variability [7]. A recent randomized controlled trial (RCT) comparing CT colonography with colonoscopy reported that CT colonography had significantly lower detection of high-risk sessile serrated lesions (SSLs), defined as dysplastic and/or ≥10mm, when compared with colonoscopy (0.8% in the CT colonography arm vs. 4.3% in the colonoscopy arm) [18]. Even though CT colonography does show adequate detection of CRC and large precursor lesions, incidental extracolonic findings were present in up to 69% of patients and ultimately only 3% needed definitive medical treatment [7].

CRC Screening Guidelines

Recommended CRC screening methods and screening intervals vary among guidelines (Table 1). While most guidelines recommend conducting FIT or gFOBT every year, the American College of Physicians (ACP) and the Canadian Task Force on Preventive Healthcare (CTFPHC) recommend these tests on a biennial basis [19,20]. American Society of Clinical Oncology (ASCO) recommends using FIT-DNA screening intervals from one year to three years. The US Multi-Society Task Force on Colorectal Cancer (MSTF) Tier 1 recommendation (most highly recommended) as well as American College of Gastroenterology (ACG) recommendation involve offering colonoscopy first, then annual FIT if patients decline colonoscopy [21,22]. FS is well-accepted across professional organizations but is recommended be used for screening every five to 10 years. The USPSTF, ACP, and ASCO provide a hybrid option combining FS with FIT [8,20,23]. Although ASCO supports the use of CT colonography for CRC screening, it is not recommended by the ACP or CTFPHC [19,20,23].

**Table 1:** Comparison of CRC screening guidelines in average risk, asymptomatic individuals Abbreviations: gFOBT (guaiac-based fecal occult blood test), FOBT (fecal occult blood test), y (year), FIT (fecal immunochemical test), mt-sDNA (multitarget stool DNA test), FS (flexible sigmoidoscopy), rec. (recommendation) 1 Suggested by manufacturer

Organization	Recommended screening tests and intervals	Preferred screening method	Age to start	Age to stop
US Preventive Services Task Force Davidson et al. 2021 [8]	gFOBT every 1 y FIT every 1 y mt-sDNA every 1 or 3 y^1^ Colonoscopy every 10 y CT colonography every 5 y FS every 5 y FS every 10 y + FIT every 1 y	Discussion of implementation considerations with patients may help better identify screening tests that are more likely to be completed by a given individual	45	75, 76-85 individual decision
American College of Physicians Qaseem et al. 2019 [20]	High sensitivity gFOBT every 2 y FIT every 2 y Colonoscopy every 10 y FS every 10 y + FIT every 2 y	Clinicians should select a screening test with the patient on a basis of discussion of benefits, harms, costs, availability, frequency, availability, and patient preferences	50	75, or ≤10 year life expectancy
American College of Gastroenterology Shaukat et al. 2021 [22]	High sensitivity gFOBT every 1 y FIT every 1 y mt-sDNA every 3 y^1^ Colonoscopy every 10 y CT colonography every 5 y FS every 5 y	Clinicians should offer patients the opportunity to select either a structural examination, or a high-sensitivity stool-based test, depending on patient preference and test availability	45 (qualified rec.)	75 or ≤10 years life expectancy (qualified rec.), 76-85 individual decision (qualified rec.)
US Multi-Society Task Force on Colorectal Cancer Rex et al. 2017 [21]	Divided into tiers: Colonoscopy (tier 1) every 10 y FIT (tier 1) every 1 y CT colonography (tier 2) every 5 y mt-sDNA (tier 2) every 3 y FS (tier 2) every 5-10 y Capsule colonoscopy (tier 3) every 5 y	Colonoscopy offered first, then FIT offered to patients who decline colonoscopy	50 (strong rec.), 45 for African Americans (weak rec.)	75 or <10 years life expectancy (weak rec.), consider up to 85 in persons without prior screening (weak rec.)
American Society of Clinical Oncology Lopes et al. 2019 [23]	In a maximal setting: High sensitivity gFOBT every 1 y (strong rec.) FS every 5 y (strong rec.) FS every 10 y + FIT every 1 y (strong rec.) FIT every 1 y (moderate rec.) Colonoscopy every 10 y (weak rec.) CT colonography (weak rec.) mt-sDNA (weak rec.)	High sensitivity gFOBT, FS, or FS + FIT (strong rec.)	50	75
Canadian Task Force on Preventive Health Care Bacchus et al. 2016 [19]	gFOBT every 2 y FIT every 2 y FS every 10 y (weak rec. 50-59, strong rec. 60-74) Do not recommend colonoscopy screening (weak rec.)	Primary care providers should discuss the most appropriate choice of test with patients considering patient values, preferences, and local test availability	50 (weak rec.)	74 (strong rec.)

In 2020, the European Society for Medical Oncology released updated guidelines recommending colonoscopy for CRC screening based on higher sensitivity and specificity when compared with other tests. FS may be a suitable alternative for those who refuse colonoscopy. The combination of FS with FOBTs is recommended to lower the risk of right-sided tumors [8]. Globally, the majority of screening guidelines support colonoscopy (every 10 years), FS (every five years), and FIT (every one or two years) [24]. One noteworthy difference is Saudi Arabia whose guidelines recommend FS every three years [25]. Germany recommends a hybrid option of FS every five years with gFOBT every one year [26].

Most importantly, many guidelines stress the importance of involving patient preferences, test availability, and likelihood of completion when discussing CRC screening tests with patients. Patients are more likely to participate if they are given a choice [27,28]. In 2018, the prevalence of US adults up to date with CRC screening reached 68.8%, but this remained below the Healthy People 2020 target of 70.5% [29]. CRC screening rates were the lowest among individuals aged 50-54 (50.0%), particularly those without a regular healthcare provider or health insurance [29]. 

Emerging Approaches in CRC Screening

Technologies that may improve CRC detection have gained attention in recent years. One of them is computer-aided detection (CADe), which involves the use of AI to provide real-time feedback to endoscopists. Wang et al. conducted a randomized control of CADe and reported a significant increase in adenoma detection rate (ADR) (29%) as compared to the standard colonoscopy group (20%) [30]. Notably, this increase was primarily due to detection of diminutive (<5mm) adenomas. More studies will need to be done to assess whether CADe could become a permanent part of colonoscopy.

Analysis of volatile organic compounds (VOCs) is another area of active research. VOCs are emitted gases that are the byproduct of metabolic processes and were shown to have an association with CRC [31-33]. Van Keulen et al. conducted a study on VOC breath patterns as a potential tool for CRC screening. The models yielded a 95% sensitivity and a 64% specificity for detecting CRC, and a 79% sensitivity and a 59% specificity for detecting advanced adenomas [34]. While the current model’s low specificity raises concerns for its applicability in screening, exhaled VOCs are still an attractive option as it may still be considered in patients who are aversive to currently available screening methods.

Another new development is the use of the QCancer® calculator. One recent study suggested to forgo screening in individuals with <3% 15-year risk of developing CRC, as determined by the QCancer® calculator [35]. While risk-based screening approaches are unlikely to significantly change the landscape of CRC screening in the US in the immediate future, further investigations will be anticipated. 

Age to start screening

CRC diagnosed before the age of 50 is classified as early-onset colorectal cancer (eoCRC). The incidence of eoCRC has increased by 51% since the mid-1990s [36]. In the US, an analysis of SEER data from 2000-2015 found a significantly increased incidence of CRC from 49 to 50 years of age (34.9 to 51.0 cases per 100,000) [37]. Based on current trends, it is estimated that the incidence rates for colon and rectal cancer will increase by 27.7% and 46.0% respectively for patients aged 35-49 years old by 2030 [38]. This is in contrast with patients over 50, in which incidence of CRC is declining overall in the last 20 years [39].

The majority of newly diagnosed eoCRC are left-sided cancers [40]. A case series conducted by Myers et al. showed that 94% of patients diagnosed with eoCRC were symptomatic at the time of diagnosis. Fifty-three percent of the patients had an advanced stage CRC [40]. This study reinforces previous independent findings by Teng et al. who demonstrated that younger patients <50 years of age with CRC have higher rates of advanced disease at time of diagnosis [41]. While select groups of patients are at higher risk of eoCRC, such as those with inflammatory bowel disease or hereditary cancer conditions, the proportion of eoCRC attributable to these conditions is relatively small. Seventy to 85% of eoCRC are sporadic [42]. This prompted researchers to suggest that eoCRC is an independent disease from the older adult counterpart. Molecular studies are ongoing and have yet to clarify its pathogenesis [43].

Due to the observed increase in eoCRC incidence, the age to begin CRC screening has been debated recently. The ACS (2018) and USPSTF (2020) revised their guidelines to recommend starting to screen for CRC at the age of 45 (Table 1) [5,44]. The MSTF (2017) recommended to start screening at 45 years for African Americans only due to increased risk factors [21,45].

Several independent software simulation models have reinforced the expected benefit in earlier screening. The Cancer Intervention and Surveillance Modeling Network (CISNET) chartered three different models (SimCRC, CRC-Spin, and MISCAN-Colon) that simulated disease progression in large cohorts of people. Two models (SimCRC and CRC-Spin) immediately supported starting colonoscopies at 45 with beneficial outcomes of life-years gained (YLG) [46]. The third model, MISCAN-Colon, was based on epidemiologic data from 1975-1979 and did not factor in a rising incidence of eoCRC during the last few decades. The ACS requested that the MISCAN-Colon model be re-simulated with current epidemiologic data provided by Peterse et al, which resulted in proof of benefits for earlier screening [47].

Some have argued against the conclusions made by these three simulations. These models assume perfect compliance with screening, follow-up, and surveillance. Younger patients have lower adherence rates to screening [48]. Efforts need to be made to increase clinician awareness about symptoms and their clinical importance in younger populations. More epidemiologic studies are needed to further describe eoCRC.

Age to stop screening

Current guidelines from the USPSTF recommend discontinuing CRC screening in average risk adults at age 75 [43]. Beyond age 75, there is more controversy and less evidence with regards to stopping CRC screening. Ultimately, the decision should be made based on patient’s comorbidities, screening history, overall life expectancy, and personal preferences [43]. Considering this, many patients aged 76-85 and a select number of patients aged 85 and older could receive benefit from CRC screening.

Post-colonoscopy/polypectomy surveillance guidelines

In 2020, the MSTF updated its post-colonoscopy/polypectomy surveillance recommendations to be increasingly personalized (Table 2) [49,50]. These guidelines assume a high-quality baseline colonoscopy and exclude populations with high CRC risk.

**Table 2:** Comparison of MSTF post-colonoscopy/polypectomy surveillance intervals for average risk adults from 2020 and 2012 Updates in the 2020 guidelines are bolded. 2020 adenoma guidelines are all strong recommendations, and serrated polyp guidelines are all weak recommendations unless otherwise noted. Abbreviations: y = year, rec= recommendation, m = month, TA = tubular adenoma, SSL = sessile serrated lesion.

Baseline High-Quality Colonoscopy Findings	2020 [49]	2012 [50]
Adenomas		
Normal	10 y	10 y
1-2 Tubular adenomas <10 mm	7-10 y	5-10 y
3-4 Tubular adenomas <10 mm (weak rec.)	3-5 y	3-10 TA: 3 y
5-10 Tubular adenomas <10 mm	3 y	
>10 Adenomas on single examination	1 y + Genetic testing	>10 Adenomas: <3 y
Any adenoma ≥10 mm (weak rec.)	3 y	≥1 tubular adenomas ≥10 mm: 3 y, ≥1 villous adenomas: 3 y
Any adenoma with tubulovillous/villous histology	3 y	
Any adenoma with high grade dysplasia	3 y	3 y
Piecemeal resection of adenoma ≥20 mm	6 m, 1 y, 3 y	None
Serrated Polyps		
≤20 Hyperplastic polyps in rectum/sigmoid colon <10 mm (strong rec.)	10 y	Hyperplastic polyps in rectum/sigmoid colon <10 mm: 10 y
≤20 Hyperplastic polyps proximal to sigmoid colon <10 mm	10 y	None
1-2 SSLs <10 mm	5-10 y	SSL(s) present <10 mm with no dysplasia: 5 y
3-4 SSLs <10 mm	3-5 y	
5-10 SSLs <10 mm	3 y	
Hyperplastic polyp ≥10 mm	3-5 y	None
SSL ≥10 mm	3 y	3 y
SSL with dysplasia	3 y	3 y
Traditional serrated adenoma	3 y	3 y
Piecemeal resection of SSL ≥20 mm (strong rec.)	6 m, 1 y, 3 y	None

This guideline update for one to two adenomas <10 mm is noteworthy because the rate of advanced neoplasia in patients with these lesions is ≤2% higher than patients with normal colonoscopy [51]. If piecemeal resection of an adenoma ≥20 mm is performed, the surveillance colonoscopy should be in six months, one year, then three years [49]. For patients with baseline adenoma findings, specifically one to four tubular adenomas <10 mm, if the first surveillance yields a normal colonoscopy, the interval until second surveillance is 10 years [49]. Adenoma greater than 10 mm, tubulovillous/villous histology, high grade dysplasia or five to 10 adenomas with a normal colonoscopy at first surveillance warrant a five-year interval. Genetic testing may be considered for patients with >10 adenomas or >10 lifetime adenomas [49].

**Figure 1:**
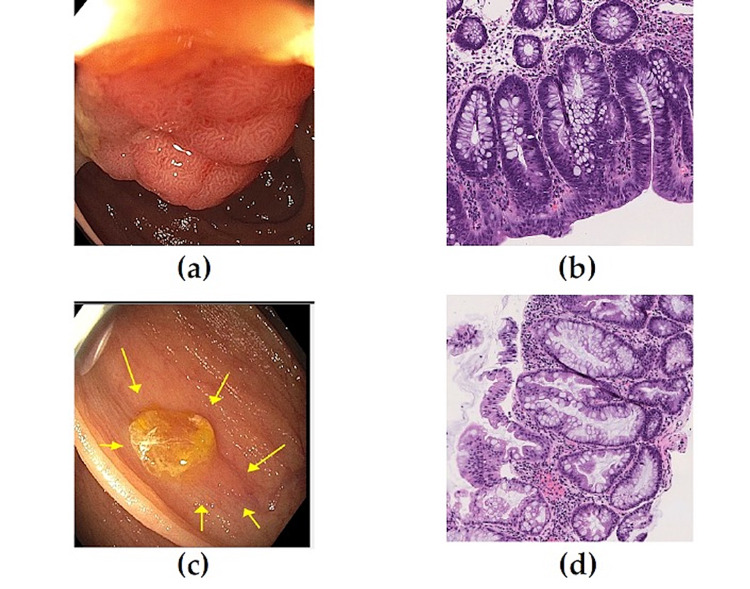
Tubular adenoma versus sessile serrated lesion (SSL) A: Large tubular adenoma, pathology showed high grade dysplasia; B: High power magnification of a tubular adenoma shows proliferating, crowded, hypercellular colonic crypts located at the surface. Nuclei are elongated, hyperchromatic, pseudostratified and retain basal orientation; C: SSL with mucus cap partially removed. As with most of serrated polyps, the margins can be quite indistinct; D: High power magnification of a SSL showing characteristic morphology with a flat growth pattern, dilation and serration of the epithelium extending to crypt bases often resulting in branched crypts. (H&E; 20X).

Serrated polyps are often difficult to detect endoscopically and yield a different pathology when compared with adenomas (Figure 1). The World Health Organization divides serrated polyps (SP) into hyperplastic polyps (HP), SSLs and traditional serrated adenomas (TSA) [52]. The new recommendations for follow-up after removal of serrated polyps are more detailed. If piecemeal resection of an SSL ≥20 mm is performed, surveillance colonoscopies are recommended at six months, one year, then three years [47]. Note that the 2012 guidelines recommended a <1-year interval for repeat colonoscopy in patients with an adenoma/SSL removed (no size specified) by piecemeal resection [50].

The ability of an endoscopist to perform a high-quality colonoscopy has been measured by their adenoma detection rate (ADR). This often results in what Gupta et al. calls the "adenoma detector paradox" [49]. Many patients have multiple (one to four) small adenomas detected and are thus categorized into the earliest possible surveillance interval even though their risk for CRC after a high-quality colonoscopy is potentially low. This issue indicates a need for more research on the outcomes of multiple adenomas and potential reconsideration of ADR as the primary quality metric.

High-quality colonoscopy

The quality of colonoscopy and the rate of post-colonoscopy colorectal cancers are closely correlated [53]. Rex et al. delineated “Quality Indicators for Colonoscopy” to include ADR, adequate bowel preparation and complete polyp resection [16]. The consensus has been that the primary indicator to determine an endoscopist’s ability to discriminate between lesions in the colon is the ADR (≥30% in men, ≥20% in women) [16]. However, ADR as a quality indicator can reward the “one and done” approach, allowing for manipulation of the system [16]. Alternatives to ADR include polyp detection rate and adenoma per colonoscopy [16,49].

**Figure 2:**
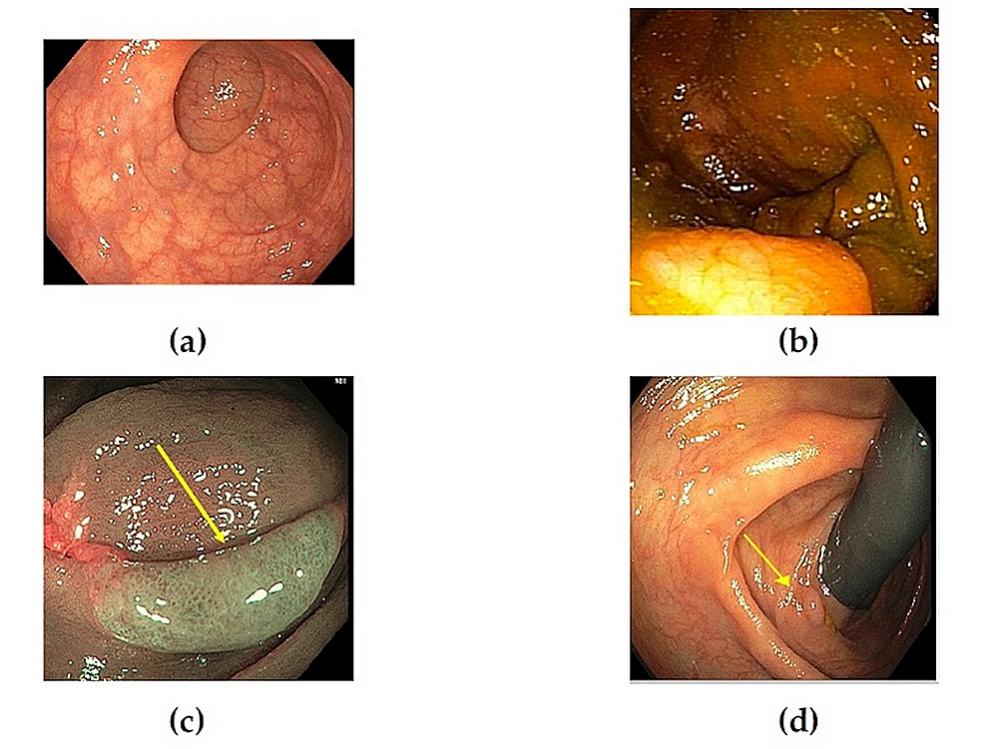
Bowel Preparation and Technique A: Excellent/optimal bowel preparation; B: Inadequate bowel preparation with adherent mucus limiting the ability to find polyps (flat); C: Serrated polyp (indicated by arrow) visualized with narrow band imaging; D: Retroflexion in the right colon (polyp on proximal side of haustral fold indicated by arrow).

Bowel preparation should be adequate enough to consistently allow detection of polyps >5 mm after suctioning leftover stool; the benchmark is 85% adequate bowel rate in clinical practice (Figure 2a) [16,50]. Most clinical practices use descriptive terms such as “excellent/optimal”, “good”, “fair”, “poor”, as opposed to a standardized scoring system which poses difficulties in conducting large-scale research [54]. Inadequate bowel preparation reduces the efficacy of screening (Figure 2b) and can reduce detection of early adenomas [55]. The timing of bowel preparation regimes is evolving. Splitting regimes involve taking half of the bowel cleansing dose the day before and the taking the other half the morning of the procedure; this method can lead to improved patient satisfaction, compliance, and ADR [56]. Recently, same-day preparation regimen in the context of afternoon colonoscopies has been shown to be as effective as split regimen (10 studies, 1807 patients, 85.3% same-day, 86.3% split group rates of adequate cleansing) [57].

Technique Updates: Narrow Band Imaging, Retroflexion and Second Assessment in the Right Colon

In contrast to typical white-light endoscopy (WLE), narrow band imaging (NBI) is a blue light (415+/-30 nm) technology that improves visualization of mucosal structures by making superficial microcapillaries appear darker (Figure 2c) [58]. The question of NBI’s efficacy in improving ADR is still in contention. Although a recent meta-analysis found an increase in adenoma detection when using high definition NBI versus high definition WLE [59], the European Society of Gastrointestinal Endoscopy concluded that the use of NBI may only lead to a marginal improvement in ADR [60]. NBI may be superior to WLE in detection of SSLs due to the irregularity of blood vessel pattern in such lesions, however more studies are needed for confirmation [61].

Retroflexion is a maneuver in which the colonoscope makes a U-turn, allowing better visualization of the proximal sides of the haustral folds (Figure 2d) [62]. This technique has been suggested for use in the right colon due to the low efficacy of conventional colonoscopy in prevention of proximal CRC [62]. A 2017 meta-analysis found that retroflexion detected 17% of adenomas in the right colon that would have been missed with conventional colonoscopy [63]. Second examination of the right colon using has been shown to improve ADR in the right colon by a very similar amount and may be preferred over retroflexion because it is an easier maneuver to perform [64].

## Conclusions

CRC continues to be a predominant cancer and cause of death worldwide. As our understanding of the course of this disease evolves, timely screening becomes very important. Our goal is to have this review shed some light on the new important changes in guidelines and expanded on the data behind those changes. We also discussed controversies concerning CRC screening methods and emerging new techniques that are currently being investigated. Our hope is that this review will prompt more research to be done to further elucidate the controversies and the new approaches discussed.
